# Anaphylaxis with Elevated Procalcitonin Mimicking Sepsis: A Literature Review and Report of Two Cases

**DOI:** 10.3390/jcm14030785

**Published:** 2025-01-25

**Authors:** András Bánvölgyi, Kende Lőrincz, Mehdi Boostani, Efrat Bar-Ilan, Bernadett Hidvégi, Márta Medvecz, Norbert Kiss, Norbert M. Wikonkál

**Affiliations:** 1 Department of Dermatology, Venereology and Dermatooncology, Semmelweis University, 1085 Budapest, Hungary; banvolgyi.andras@semmelweis.hu (A.B.); lorincz.kende@semmelweis.hu (K.L.); mehdi_parsii@yahoo.com (M.B.); hidvegi.bernadett@semmelweis.hu (B.H.); medvecz.marta@semmelweis.hu (M.M.); wikonkal.norbert@semmelweis.hu (N.M.W.); 2 Pediatric Dermatology Department, Heim Pál National Children’s Institute, 1089 Budapest, Hungary; efratbarilan@gmail.com; 3 Pediatric Dermatology Unit, Dermatology Department, Sheba Medical Center, Ramat Gan 5262160, Israel

**Keywords:** procalcitonin, anaphylaxis, sepsis, sulfamethoxazole, trimethoprim, anaphylactic shock, misdiagnosis, allergy, allergic drug reaction, differential diagnosis

## Abstract

**Objectives**: This review examines the role of serum procalcitonin (PCT) as a diagnostic marker for sepsis and its potential implications in anaphylaxis. Elevated PCT levels, often associated with bacterial infections, can complicate diagnosis when seen in anaphylaxis, especially when clinical features overlap with sepsis. **Methods**: We conducted a literature review on PCT in anaphylaxis to highlight key patterns and present two cases of anaphylactic shock initially misdiagnosed as sepsis due to elevated PCT levels. **Results**: The review supports that elevated PCT can occur in anaphylaxis, stressing the need for thorough patient history and symptom evaluation. In both cases, elevated PCT led to initial sepsis diagnoses, but further investigation identified anaphylaxis triggered by sulfamethoxazole/trimethoprim (SMX/TMP). **Conclusions**: These findings emphasize the need for considering anaphylaxis in differential diagnoses when elevated PCT levels are observed. Increased awareness of PCT’s potential association with allergic drug reactions is essential to ensure timely recognition, avoid diagnostic delays, and improve patient outcomes.

## 1. Introduction

Procalcitonin (PCT) is widely recognized as a biomarker for sepsis and bacterial infections, aiding clinicians in distinguishing bacterial from non-bacterial inflammatory states [1]. Both the FDA and EMA have approved the use of PCT assays to guide antibiotic therapy in sepsis and lower respiratory tract infections, underscoring its critical role in clinical decision-making [2,3]. However, the potential role of elevated PCT levels in non-infectious conditions such as anaphylaxis has gained increasing attention in recent years [4]. Anaphylaxis is a severe systemic hypersensitivity reaction that can present with overlapping clinical and laboratory features of sepsis, including fever, tachycardia, hypotension, and elevated inflammatory markers such as C-reactive protein (CRP) and white blood cell (WBC) count [5,6,7]. This overlap poses significant diagnostic challenges, particularly in critically ill patients where prompt and accurate diagnosis is essential for effective management.

Although the exact mechanism of PCT production during anaphylaxis is not fully understood, it may be linked to the cytokine cascade that plays a central role in the pathophysiology of this severe hypersensitivity reaction. Anaphylaxis involves the activation of mast cells and basophils, which release histamine and other mediators, initiating a complex inflammatory response. This response is further amplified by the release of cytokines such as interleukin (IL)-6, IL-10, and tumor necrosis factor-alpha (TNF-α), which have been shown to be elevated in severe anaphylactic reactions. Specifically, IL-6 and IL-10 correlate with the presence of hypotension, a critical feature of anaphylaxis [8].

Procalcitonin’s involvement may arise from its regulation at the gene level. The calc-1 gene, responsible for PCT production, is typically expressed in thyroid C cells. However, during systemic inflammatory states, such as bacterial infections or possibly anaphylaxis, non-thyroid tissues may express PCT through alternative signaling pathways. This aberrant expression could be influenced by the inflammatory milieu, with cytokines like IL-6 serving as potential triggers for PCT production. While PCT is primarily known as a precursor for calcitonin, during such pathological conditions, its secretion into the bloodstream may serve as a nonspecific marker of systemic inflammation rather than its traditional role in calcium homeostasis [9].

Furthermore, in anaphylaxis, mediators such as histamine and mast cell tryptase peak quickly, while cytokines like IL-6 and TNF receptor I rise later, aligning temporally with the potential activation of alternative pathways for PCT production. It is plausible that the cytokine-driven upregulation of PCT mirrors its behavior in bacterial infections, where IL-6 acts as a synergic activator of the Calc-1 promoter, driving PCT transcription. These molecular events underscore a potential link between the cytokine cascade in anaphylaxis and PCT elevation, warranting further investigation into the mechanistic role of PCT in non-infectious inflammatory states [8].

Despite growing recognition of elevated PCT levels in anaphylaxis, this phenomenon remains underreported, with limited literature available to guide clinicians. In particular, drug-induced anaphylaxis, such as that caused by sulfamethoxazole/trimethoprim (SMX/TMP) [10], can mimic sepsis due to its association with elevated PCT [11].

Understanding this relationship is critical to avoid misdiagnosis and unnecessary antibiotic administration, which can lead to increased healthcare costs, potential side effects, and the development of antimicrobial resistance. Advances in diagnostic technology [12,13,14,15,16], such as improved biomarker assays [17] and the integration of data-driven decision-support tools [18], hold significant promise for addressing these challenges. By refining the interpretation of biomarkers like PCT and integrating them with clinical and contextual data, these technologies have the potential to enhance diagnostic accuracy, reduce diagnostic uncertainty, and guide more targeted therapeutic interventions.

Given the diagnostic complexity in differentiating between sepsis and anaphylaxis, especially in critically ill patients, understanding the potential for elevated PCT in non-infectious conditions is essential. Clinicians must be cautious when interpreting elevated PCT levels, particularly in the context of anaphylactic reactions, to avoid misdiagnosis and unnecessary antibiotic use. Early recognition of anaphylaxis, particularly drug-induced reactions like those caused by SMX/TMP, is critical to ensure timely, appropriate treatment, prevent diagnostic delays, and reduce unnecessary healthcare costs. Here, we present two unpublished cases of SMX/TMP-induced anaphylaxis characterized by significantly elevated PCT levels, highlighting the diagnostic complexity in distinguishing between infectious and non-infectious causes of systemic inflammation. These cases are complemented by a comprehensive review of the literature, which examines the clinical presentation, overlapping features with sepsis, and the associated diagnostic challenges posed by elevated PCT in the context of anaphylaxis. Furthermore, this review provides insights into best practices for managing such cases, including the importance of integrating clinical findings, biomarker interpretation, and a thorough medication history to avoid misdiagnosis and ensure appropriate treatment strategies.

## 2. Case Presentations

### 2.1. Case 1

**Figure 1:**
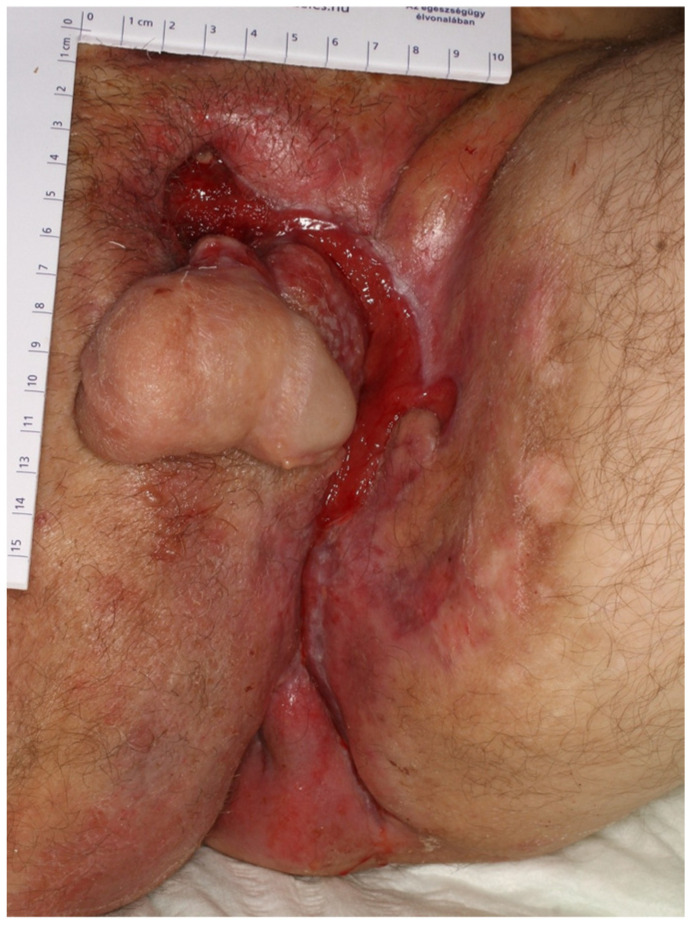
Clinical presentation of a 39-year-old male patient with extensive genital hidradenitis suppurativa prior to treatment with sulfamethoxazole/trimethoprim (SMX/TMP). The patient presented with multiple deep ulcers in the suprapubic region, perigenital area, and left inguinal and gluteal folds, suspected to be infected. Bacterial cultures confirmed the presence of *Proteus mirabilis*, *Staphylococcus aureus*, and Group G Streptococci. This image highlights the severity and extent of the patient’s lesions, which contributed to the diagnostic challenges and the decision to initiate empirical antibiotic therapy. Subsequent episodes of systemic reactions were linked to SMX/TMP, emphasizing the importance of recognizing drug-induced anaphylaxis in complex clinical scenarios.

A 39-year-old male with a history of hidradenitis suppurativa and known drug allergies to amoxicillin, ceftriaxone, and moxifloxacin was admitted to the hospital with extensive, deep ulcers located in the suprapubic region, around the penis, and in the left inguinal and gluteal folds (Figure 1). The patient’s ulcers were suspected to be infected, and bacterial cultures confirmed the presence of Proteus mirabilis, Staphylococcus aureus, and Group G Streptococci. Initial laboratory tests revealed a WBC count of 5.43 × 10^9^/L and a CRP level of 57.4 mg/L, both of which indicated an ongoing inflammatory process associated with the infection. Based on the antibiogram results, SMX/TMP was initiated as part of the empirical antibiotic therapy.

Several hours after the first dose of SMX/TMP, the patient developed a high fever of 39 °C accompanied by chills. His condition worsened the following day, when he became increasingly lethargic, hypotensive, and tachycardic, prompting further investigation. Laboratory results at that time revealed a significantly elevated serum PCT level of 303 ng/mL, alongside an increase in his WBC count to 9.55 × 10^9^/L and a rise in CRP to 125.4 mg/L. Given the severity of his clinical presentation, sepsis was suspected, and the patient was transferred to the intensive care unit (ICU) for closer monitoring and aggressive treatment. In the ICU, his antibiotic regimen was switched to tigecycline and clindamycin, which led to a gradual stabilization of his condition. Over the next few days, his PCT levels steadily normalized, and his clinical status improved.

Seven days later, SMX/TMP was reintroduced to treat his ongoing infection. However, the patient rapidly developed a recurrence of fever, and his PCT levels spiked again, reaching 50.91 ng/mL. This prompted an immediate discontinuation of the antibiotic. Further diagnostic workup revealed an elevated serum tryptase level, confirming the diagnosis of an anaphylactic reaction. Retrospective analysis of the patient’s clinical history revealed that similar episodes of systemic reaction had occurred exclusively during SMX/TMP therapy. This pattern strongly suggested recurrent drug-induced anaphylaxis, reinforcing the need for avoidance of SMX/TMP in this patient in the future.

### 2.2. Case 2

**Figure 2:**
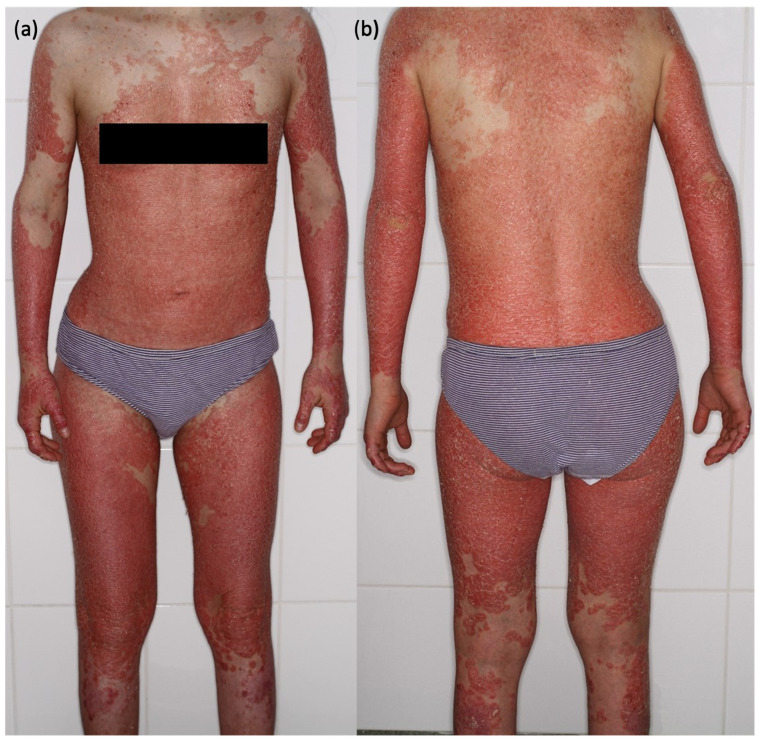
Clinical features of a 21-year-old female patient with Down syndrome, chronic plaque psoriasis, and psoriatic arthritis who was hospitalized for erythroderma. The condition, characterized by widespread erythema and scaling, is displayed in (**a**) anterior and (**b**) posterior views. This patient presented with generalized inflammatory skin changes secondary to her underlying diseases. Following tooth extraction and initiation of sulfamethoxazole/trimethoprim (SMX/TMP) as prophylaxis, she developed acute systemic reactions, later confirmed as drug-induced anaphylaxis. The clinical image captures the severity of her baseline inflammatory skin condition, which compounded diagnostic uncertainty during her acute reaction.

A 21-year-old female with Down syndrome, chronic plaque psoriasis, and psoriatic arthritis was hospitalized for erythroderma, a severe and widespread inflammatory skin condition characterized by generalized erythema and scaling (Figure 2). Her medical history was notable for multiple drug allergies, including penicillin, amoxicillin, cefuroxime, ciprofloxacin, and clarithromycin, complicating her management. Laboratory investigations upon admission revealed an elevated CRP level of 52.5 mg/L, likely reflective of the extensive inflammation caused by her underlying skin and joint diseases.

During her hospitalization, she underwent an apical tooth extraction for chronic periodontitis, after which SMX/TMP was prescribed as prophylactic antibiotic therapy. Shortly after the first dose, she experienced an acute systemic reaction, presenting with a high-grade fever of 40 °C, chills, profound malaise, hypotension, tachycardia, and dizziness. Laboratory findings revealed a marked increase in inflammatory markers, with CRP rising to 102.8 mg/L and PCT reaching 5.57 ng/mL. Imaging studies, including chest X-ray and abdominal ultrasound, were unremarkable, showing no evidence of an infectious source.

Although the initial clinical presentation raised concerns for sepsis, the temporal association of symptoms with the administration of SMX/TMP, along with her significant history of drug allergies, prompted a reassessment of the diagnosis. Drawing on lessons learned from a previously reported case (Case 1), a strong suspicion of an anaphylactic reaction was established. Prompt discontinuation of SMX/TMP and administration of intravenous methylprednisolone and chloropyramine led to the rapid resolution of her symptoms. Subsequent monitoring showed normalization of inflammatory markers, with her PCT level declining to 0.15 ng/mL, further supporting the diagnosis of drug-induced anaphylaxis.

## 3. Literature Review

A total of five cases of drug-induced anaphylaxis associated with elevated PCT levels were identified, providing valuable insights into the clinical features, diagnostic challenges, and outcomes of these events (Table 1). Patient ages ranged from 52 to 74 years, with a mean age of 62.3 ± 10.3 years, and all cases involved female patients. Most cases reported underlying medical conditions, including hypertension, diabetes mellitus, and malignancies, while allergies were noted in only one patient. The causative drugs included amoxicillin-clavulanate, hydrochlorothiazide, risedronate sodium, and SMX/TMP. Onset of symptoms varied from minutes after the first dose to several days post-administration, with all cases presenting with systemic manifestations such as wheezing, fever, nausea, rash, dyspnea, and hypotension. Laboratory findings frequently revealed elevated inflammatory markers, including CRP and leukocytosis, as well as significantly increased PCT levels, which ranged from 9.36 ng/mL to 329 ng/mL, with a mean of 112.7 ± 122.2 during the initial episodes. In cases involving drug reintroduction, PCT levels remained elevated but were comparatively lower than during the initial episodes. Treatment protocols included epinephrine, corticosteroids, antihistamines, and supportive therapy, with one case requiring albuterol nebulization. Broad-spectrum antibiotics were often administered initially but were subsequently discontinued after confirming non-infectious etiologies. All patients achieved complete recovery following appropriate intervention. Elevated PCT levels in anaphylaxis have been previously documented, as summarized in Table 1.

## 4. Discussion

Our cases underscore the potential for elevated PCT levels to mimic sepsis in the setting of drug-induced anaphylaxis, which poses a significant diagnostic challenge. PCT has long been recognized as a reliable biomarker for bacterial infections and sepsis, aiding clinicians in differentiating between infectious and non-infectious causes of systemic inflammation. However, as demonstrated in these cases, elevated PCT levels cannot always be used to reliably distinguish between infectious and non-infectious etiologies. Drug-induced anaphylaxis, particularly in response to certain medications, can cause systemic inflammation that leads to a rise in PCT levels, despite the absence of infection [24]. This highlights the crucial point that PCT levels should not be used in isolation for diagnostic decision-making, especially when clinical presentation and laboratory findings suggest infection, but definitive confirmation of an infectious source is lacking.

Both cases also emphasize the critical importance of obtaining a detailed and com-prehensive medication history in patients presenting with acute systemic symptoms following drug administration. A careful review of recent medication use, including those known to cause hypersensitivity reactions, can provide valuable clues that help clinicians differentiate between sepsis and non-infectious mimics like drug-induced anaphylaxis. This becomes even more critical in cases where the clinical symptoms overlap significantly, such as fever, tachycardia, hypotension, and elevated inflammatory markers [25]. Recognizing these patterns and maintaining a high index of suspicion for anaphylaxis, despite the presence of high inflammatory markers, can lead to quicker, more accurate diagnoses and prevent unnecessary antibiotic treatments or other invasive interventions.

Ultimately, these cases serve to emphasize the importance of integrating clinical judgment, laboratory findings, and a thorough patient history when interpreting elevated PCT levels. Clinicians must not rely solely on biomarkers for diagnosis but consider the full clinical picture to guide decision-making. Future research should focus on further elucidating the mechanisms behind PCT elevation in non-infectious conditions and developing evidence-based guidelines that can enhance diagnostic accuracy in these complex, challenging scenarios. Understanding the nuances of how biomarkers behave in drug-induced hypersensitivity reactions will be essential for refining diagnostic protocols and improving patient outcomes in similar cases.

These reported cases reveal a spectrum of PCT elevations, which vary significantly depending on the severity of the anaphylactic reaction, the timing of the PCT measurement, and individual patient factors. In some instances, PCT levels may rise modestly, while in others, the increase can be much more pronounced. For example, Mirijello et al. [19] 19 described a 52-year-old female patient who developed anaphylaxis after exposure to amoxicillin–clavulanate, with a PCT level of 9.36 ng/mL. The patient’s clinical presentation included classic signs of anaphylaxis such as wheezing, a skin rash, and loss of consciousness, along with elevated CRP and transient ECG changes suggestive of cardiovascular stress. Despite the elevated PCT levels, which could easily have been mistaken for a marker of bacterial infection, the patient responded well to treatment with epinephrine and corticosteroids, recovering fully. This case is comparable to our second reported case, where a mild elevation in PCT was observed in the context of SMX/TMP-induced anaphylaxis.

In both cases, the elevated PCT was transient and likely reflected the acute inflammatory response associated with the anaphylactic reaction rather than an underlying infection. These cases emphasize that the degree of PCT elevation is not necessarily indicative of the severity of anaphylaxis or infection. Factors such as the timing of PCT measurement, individual patient characteristics (such as comorbidities and immune response), and the specific drug involved can all influence PCT levels. As such, clinicians must exercise caution when interpreting PCT results in the context of acute allergic reactions, as high PCT levels alone should not automatically suggest a bacterial origin. Recognizing the transient nature of PCT elevation in these cases and considering other diagnostic clues, such as patient history and clinical presentation, is essential for accurate diagnosis and appropriate management.

In contrast, Al Hillan et al. [20] reported a 71-year-old female patient who experienced an anaphylactic reaction to hydrochlorothiazide. In this case, the patient’s PCT level, initially undetectable upon admission, rose dramatically to 329 ng/mL within just 9 h. This rapid and significant increase in PCT levels mirrors the extreme elevation observed in our first case, where a similarly severe anaphylactic episode resulted in a marked rise in PCT. The swift and substantial spike in PCT in these cases suggests that severe anaphylactic reactions can lead to a pronounced and rapid inflammatory response, potentially triggering a cytokine-driven cascade that results in elevated PCT levels. The timing and magnitude of PCT elevation in these cases support the idea that severe, acute reactions, particularly those with significant systemic involvement, can lead to a rapid increase in this biomarker, further complicating the clinical picture and potentially leading to confusion with sepsis or other inflammatory conditions. This underscores the need for clinicians to carefully evaluate the clinical context when interpreting PCT levels, especially in patients with known drug allergies or those experiencing anaphylaxis, as it highlights the complexity of distinguishing between infectious and non-infectious causes of inflammation.

Similarly, Kim et al. [21] described a 74-year-old female patient who experienced anaphylaxis due to risedronate sodium. Her PCT levels rose to 168 ng/mL during the first episode and to 42.3 ng/mL upon re-exposure. These findings align with the recurrent PCT elevations documented in our first case and underscore the importance of avoiding re-exposure to suspected allergens.

Mann et al. [22] reported a case of SMX/TMP-induced anaphylaxis in a 52-year-old female patient, with a PCT level of 29 ng/mL. This case reflects the moderate elevations observed in our second patient, further supporting the association between SMX/TMP and PCT elevations in anaphylaxis. Additionally, Hounoki et al. [23] described a patient with autoimmune disease who experienced PCT elevations of 28.12 ng/mL during the first episode of SMX/TMP-induced anaphylaxis and 14.28 ng/mL during re-exposure. These findings are consistent with the recurrent nature of anaphylactic reactions noted in our first case.

In another case reported in 2024, it was observed that elevated PCT levels can fluctuate with piperacillin/tazobactam use, returning to normal after the drug is discontinued, coinciding with the patient’s clinical improvement [26].

From these cases, several key takeaways emerge. First, while PCT elevations vary in magnitude, even mild increases, as seen in our second case, can signify early anaphylaxis. Conversely, extreme PCT elevations, as documented in our first case and in the reports by Al Hillan et al. [20] and Kim et al. [21], are indicative of severe reactions. Second, recurrent reactions, as observed in Hounoki et al. [23] and our first case, highlight the importance of avoiding re-exposure to the causative drug. Finally, the overlap between anaphylaxis and sepsis, particularly in cases with elevated PCT, poses a significant diagnostic challenge. Misdiagnosing anaphylaxis as sepsis may delay withdrawal of the causative medication and appropriate treatment, potentially worsening patient outcomes.

Our cases, along with the existing literature, suggest that PCT elevations in anaphylaxis may be a result of a cytokine storm triggered by the widespread systemic inflammation that occurs during these reactions. However, the exact mechanisms underlying this phenomenon are still not fully understood and require further investigation. It is hypothesized that the acute inflammatory response associated with anaphylaxis can stimulate the release of proinflammatory cytokines, which, in turn, may drive PCT production as part of the body’s response to severe hypersensitivity reactions [8]. This mechanism, though plausible, has yet to be clearly defined and warrants further research to explore the specific pathways involved. The cytokine storm during anaphylaxis involves the release of cytokines such as IL-6, TNF-α, and IL-1β, which stimulate the liver to produce PCT as part of the acute-phase response. In addition to cytokine release, endothelial dysfunction and vascular permeability during anaphylaxis can exacerbate the inflammatory cascade, contributing to PCT production [27]. Mast cell degranulation, central to anaphylaxis, releases various proinflammatory mediators that may further amplify systemic inflammation and elevate PCT levels. Complement system activation could also play a role in promoting cytokine release and, subsequently, PCT production. While PCT is traditionally used as a marker for bacterial infection, its elevation in anaphylaxis likely reflects a severe, non-infectious inflammatory response, highlighting the need for additional research to clarify the underlying mechanisms and temporal dynamics of PCT release in these reactions. This exploration could improve both diagnosis and treatment strategies, especially in differentiating between infection and the inflammatory response associated with anaphylaxis.

Clinicians should be aware of the potential for PCT elevation in anaphylaxis, particularly in patients who experience an abrupt onset of symptoms following drug administration. Since elevated PCT levels are often interpreted as indicative of bacterial infections or sepsis [28], this may lead to misdiagnosis if not carefully considered. Recognizing that PCT elevation can occur in the absence of infection, especially in the context of drug-induced anaphylaxis, is crucial for appropriate clinical management. Promptly identifying the source of the anaphylactic reaction and discontinuing the causative drug can be life-saving, as it prevents further escalation of symptoms and facilitates recovery. Given the challenges in distinguishing between infectious and non-infectious causes of systemic inflammation, greater awareness and research into the pathophysiology of PCT elevation in anaphylaxis will enhance diagnostic accuracy and guide better treatment decisions.

## 5. Conclusions

Anaphylaxis with elevated PCT is a diagnostic challenge that can mimic sepsis. Our cases, alongside the literature, highlight the need for heightened awareness among clinicians to recognize this phenomenon. SMX/TMP, which is frequently implicated, requires special caution. Further studies are necessary to understand the mechanisms and improve diagnostic accuracy.

## Figures and Tables

**Table 1:** Overview of reported cases of drug-induced anaphylaxis with elevated procalcitonin (PCT) levels, summarizing patient characteristics, medical history, causative drugs, timing of symptom onset, clinical features, laboratory findings, PCT levels during initial and subsequent episodes, treatment approaches, and outcomes. This table highlights the diversity of clinical presentations, laboratory findings, and therapeutic strategies used in the management of these cases, while illustrating the diagnostic challenges posed by elevated PCT levels in non-infectious conditions such as anaphylaxis.

No	Case	Age	Sex	Past Medical History	Allergies	Causative Drug	Onset of Symptoms	Presenting Clinical Symptoms and Laboratory Findings	PCT (ng/mL) Episode 1 (RR < 0.5 ng/mL)	PCT (ng/mL) Episode 2 (After Re-introducing the Causative Drug)	Treatment	Outcome
1	Mirijello et al., Italy, 2020 [19]	52	F	None	None	Amoxicillin- clavulanate	Minutes after the first dose	Wheezing, skin rash, dizziness, loss of consciousness, hypertension, tachycardia, and tachypnoea. Leucocytosis and high CRP. (ECG: ST-segment depression in inferior-lateral leads. Troponin was slightly high, in absence of chest pain ^#^).	9.36	-	Initially: high-dose IV epinephrine, suppurative therapy, chlorphenamine, and hydrocortisone. Later on: IV hydrocortisone and clarithromycin.	Complete recovery
2	Al Hillan et al., USA, 2019 [20]	71	F	Hypertension, type II DM and adeno-carcinoma of the lung (status post resection and chemotherapy)	Sulfa	Hydrochloro-thiazide	Minutes after the first dose	Pruritus, shortness of breath, wheezing, diaphoresis, hypotension, and tachycardia. Elevated tryptase level.	On admission: negative 3 h: 88 9 h: 329	-	Epinephrine, suppurative therapy prednisone, antihistamine, and broad-spectrum antibiotic (stopped after 1 day).	Complete recovery
3	Kim et al., South Korea, 2015 [21]	74	F	S/P gastrectomy for gastric cancer and pituitary tumor resection	N/A	Risedronate sodium	First episode: 3 days after initial dose. Second episode: minutes after reintroducing the medication.	Nausea, fever, chills, severe, hypotension and tachycardia. Elevated CRP and slightly elevated ESR.	168	42.3	First episode: norepinephrine, suppurative therapy, and received broad-spectrum antibiotics. Second episode: withdrawal of medication.	Complete recovery
4	Mann et al., USA, 2014 [22]	52	F	Hypertension and seborrheic dermatitis	N/A	SMX/TMP	Minutes after the first dose	Nausea, vomiting, hives, dyspnea, pharyngeal fullness, fever, tachycardia and hypotension. Slightly elevated CRP, elevated lactic acid and serum tryptase level	29	-	Epinephrine, suppurative therapy methylprednisolon, diphenhydramine famotidine, albuterol nebulizer and broad-spectrum antibiotic (single dose)	Complete recovery
5	Hounoki et al., Japan, 2013 [23]	N/A	N/A	Unspecified systemic autoimmune disease treated with 30 mg/d prednisolone	N/A	SMX/TMP	First episode: 12 days after initiating therapy. Second episode: minutes after reintroducing the medication.	Nausea, vomiting, fever, severe hypotension and tachycardia, elevated CRP, leukocytosis, and thrombocytopenia. (Presence of a hypercoagulable state, elevated concentrations of fibrin/fibrinogen degradation products, D-dimer, and fibrinogen monomer complex ^##^).	28.12	14.28	First episode: withdrawal of medication, 30 mg/d prednisolone and broad-spectrum antibiotic. Second episode: withdrawal of medication.	Complete recovery

CRP: C-reactive protein, DM: Diabetes mellitus, ESR: Erythrocyte sedimentation rate, F: Female, M: Male, N/A: Not available, PCT: Procalcitonin, RR: Reference range, SMX/TMP: Sulfamethoxazole/trimethoprim, USA: United States of America, Y: Years, #: Attributed to the effect of high-dose intravenous epinephrine administration, ##: The presence of a hypercoagulable state along with thrombocytopenia was attributed to the fact anaphylaxis may also result in the consumption of coagulation factors.

## Data Availability

The data that support the findings of this study are available from the corresponding author N.K. upon reasonable request.
